# Clinical Evaluation of Patients with Spinal Cord Infarction in Mashhad, Iran

**DOI:** 10.4061/2010/942417

**Published:** 2010-10-26

**Authors:** Kavian Ghandehari, Mohammad Reza Gerami Sarabi, Parham Maarufi

**Affiliations:** ^1^Neuroscience Research Center, Mashhad University of Medical Sciences, Mashhad, Iran; ^2^Department of Neurology, Ghaem Hospital, Ahmadabad Street, P.O. Box 91766-99199, Iran; ^3^Department of Orthopedic Surgery, Tabriz University of Medical Sciences, P.O. Box 8768, Tabriz, Iran

## Abstract

*Background*. Spinal Cord Infarction (SCI) is a rare and disabling disease. This hospital-based study was conducted for clinical evaluation of SCI patients in east of Iran. *Methods*. Consecutive SCI patients admitted in Ghaem hospital,Mashhad during 2006–2010 were enrolled in a prospective clinical study. Diagnosis of SCI was made by neurologists and radiologists. Demographic features, clinical syndrome, and Magnetic Resonance Imaging (MRI) findings were recorded. All of the patients underwent a standard battery of diagnostic investigations. All of the patients suspected to SCI had MRI of spinal cord at the symptomatic level of cord with a 0.5 Tesla generation, Philips NT Intra, Netherland equipment. An equal number of patients with Brain Infarction (BI) were randomly selected from our stroke registry data bank. Etiology and degree of disability were compared between these groups of patients. *Results*. Fourteen SCI patients (9 females, 5 males) with mean age 38.8 ± SD: 19.9 years were evaluated. Miscellaneous causes consisted 50% of etiologies in patients with SCI. Uncertain etiology, atherosclerosis, and cardioembolisms consisted 35.7%, 7.1%, and 7.1% of SCI causes, respectively. Distribution of etiologies was significantly different between SCI and BI patients, *X* ^2^ = 12.94, *df* = 3, *P* = .003. Difference in mean disability score at acute phase of stroke was not significant between two studied groups, *z* = 1.54, *P* = .057. Difference in mean changes of disability score at 90 days postevent was significant in two groups of patients, *z* = 2.65, *P* = .019. *Conclusion*. SCI is a rare disease with poor recovery. Distribution of etiologies of SCI patients is quite different than of BI patients.

## 1. Introduction

Compared with incidence of brain infarcts, spinal ischemic stroke remains rare, and its prevalence is not precisely known [1]. Obviously a major cause of this difference is that the spinal cord is substantially smaller than the brain [1]. Another reason of rarity of Spinal Cord Infarction (SCI) may be found in the spinal arteries which are rarely affected by atherosclerosis. Besides this, the spinal cord has a rich anastomotic network of arteries [1]. The various clinical syndromes related to spinal infarcts depend on what part of the vascular system is affected and on whether the pathologic process is acute or progressive in nature. SCI is a diagnostic puzzle with a dismal prognosis. An upsurge of interest to SCI has replaced the neglect of former years. This has been due in part to the increased accidental production of SCI by cardiovascular surgery and to the advances in Magnetic Resonance Imaging (MRI) which has allowed better visualization of the spinal cord [2]. SCI in territory of anterior spinal artery appears with abrupt neck or back pain followed with a sever flaccid paraplegia or tetraplegia and early sphincter disturbances [1, 2]. Because of spinal shock, the paraplegia may remain flaccid for several days to weeks. The upper limbs may develop motor neuron signs, and spasticity of legs may occur with hyperreflexia and extensor plantar responses. Anesthesia to pain and temperature below the level of infarct without involvement of proprioceptive and vibration sensation is present [1, 2]. SCI in territory of posterior spinal artery is very rare and involves posterior columns of the spinal cord. It presents with paresthesias and abolition of deep sensation below the level of the infarct [1, 2]. Occlusion of a central sulcal artery rarely produces small lesions in half of the spinal cord. This can present as an incomplete Brown-Sequard syndrome [2]. Total transverse SCI involves both anterior and posterior spinal artery territory and may be misdiagnosed as transverse myelitis [2]. In older patients, the sudden onset of neurologic symptoms and signs related to the spinal cord strongly suggests vascular disease. If the neurologic manifestations are attributable to a specific spinal arterial territory, the diagnosis is almost certain [2]. Vascular event in the spinal cord can be less abrupt in onset, depending mainly on the underlying mechanism and on the presence of collateral circulation [1, 2]. This is the first hospital-based clinical evaluation of patients with SCI published from Iran. 

## 2. Patients and Methods

All of the consecutive patients with SCI admitted in Ghaem hospital, Mashhad during 2006–2010 were enrolled in a prospective clinical study. The Khorasan and Mashhad stroke data bank registries are established since 2001 and prospectively register stroke patients till 2020.

Anterior spinal artery syndrome was defined as [2] (1) back or neck pain with sudden onset; (2) rapidly progressive paraplegia; (3) flaccid paraplegia that soon becomes spastic; (4) areflexia at onset, which becomes hyperreflexia with extensor plantar response; (5) sensory level for pain and temperature; (6) preserved proprioception, light touch, and vibration sense; (7) urinary incontinence; (8) painful burning dysesthesia below level of cord injury during chronic phase. Posterior spinal artery syndrome was defined as [2] (1) sensory loss for proprioception, vibration, and light touch sensation with sensory level; (2) preserved pain and temperature sensation, except at the level of the affected cord segment, where there is global anesthesia; (3) motor function preserved; (4) loss of deep tendon reflexes at the level of affected cord segment. Patients with acute onset anterior or posterior spinal artery syndrome and incomplete Brown-Sequard syndrome were assumed as SCI even without MRI evidence of SCI [1, 2]. Patients with acute transverse cord syndrome were assumed as SCI only with evidence of vascular disease in the spinal cord MRI. We excluded patients whose myelopathies were directly due to spinal cord trauma, tumor, abscess, syringomyelia or in whom there was a history of multiple sclerosis as discussed by Warlow et al. [3]. Patients with a gradual onset (longer than 24 hours) of transverse myelopathy were also excluded. All of the patients suspected to SCI had MRI of spinal cord at the symptomatic level of cord [3] with a 0.5 Tesla generation, Philips NT Intra, Netherland equipment. MRI has become the method of choice for diagnosing SCI, since it reliably excludes compressive lesions, intramedullary neoplasms, and cavitations [1, 2]. The infarcted cord can have an increased diameter on MRI. Infarcts can first be seen on *T*_2_ weighted images as high signal lesions during the acute phase [1, 2]. However during the first hours or days after the damage, even *T*_2_ image scan may remain normal [1]. All of the spinal cord MRI were performed within 24 hours postevent and reported by an experienced radiologist. A complete past medical history and neurologic examination were taken in all of the suspected SCI patients by a neurologist. An equal number of patients with Brain Infarction (BI) were randomly selected from our prospective stroke data bank registry. Random selection of 14 BI patients was done by SPSS 13 software package. All of the SCI patients and randomply selected group of BI patients underwent a standard battery of diagnostic investigations for detecting etiology of stroke [3]. This included electrocardiography, blood electrolytes, blood count and differential, coagulation profile, fasting blood sugar, lipid profile, brain CT scan, duplex sonography of supraaortic trunks, and transthoracic echocardiography. An extended coagulation profile, vasculitis profile, 24-hour Holter monitoring, transesophageal echocardiography, transcranial doppler, blood culture, Brain MRI, and angiography were performed in selected patients [3]. Etiology of SCI and BI was determined based on the Asian Stroke Criteria (ASC) [4]. The ASC was developed to detect the etiology and topography of brain infarction. The ASC was designed by stroke neurologists and approved in the University of Alberta, Canada in 2003. In the first validation step, interrater reliability of ASC was evaluated. The interrater agreement according to the ASC system is much higher than other classifications with moderate interobserver reliability. In the second validation step, the ASC has been used successfully in Middle East stroke registries [4]. Hospital-based frequency of SCI in our stroke division was calculated. Disability of SCI and BI patients was detected based on the Modified Rankin Scale (MRS) at 2 days postevent and at 90 days later [3]. Followup examinations were performed when possible, and when not, information about disability was gathered by telephone. Research project was approved by ethics committee of Ghaem hospital. An informed consent signature was taken by patient or his/her first-degree relatives. Mann-Whitney *U* test, independent *T* test, and Chi-Square test served for statistical analysis.

## 3. Results

Fourteen patients (9 females, 5 males) with SCI were admitted in Ghaem hospital during 2006–2010. Among 6802 patients (3602 females, 3200 males) with BI in our prospective stroke registry data bank, a randomly selected equal number of patients were recruited. The hospital-based frequency of SCI in our ischemic stroke patients is 0.2%. Female gender was significantly more preponderant to SCI than BI, *d* *f* = 1, *P* =  .034. Mean age of patients with BI and SCI was 64.36 ± SD: 7.53 and 38.86 ± SD: 19.95 years, respectively, which is significantly different, *t* = 4.475, *d* *f* = 26, *P *<  .001. Miscellaneous causes consisted 50% of etiologies in patients with SCI, and 21.4% of SCI patients had iatrogenic causes. These iatoigenic causes were also included within miscellaneous etiologies. Uncertain etiologies, atherosclerosis, and cardioembolisms consisted 35.7%, 7.1%, and 7.1% of SCI causes, respectively. None of our SCI or BI groups of patients had mixed or multiple causes of stroke. Atherosclerosis, cardioembolism, uncertain and miscellaneous etiologies constituted 57.1%, 21.4%, 14.3%, and 0% of causes in 14 randomly selected patients with BI. Distribution of etiologies was significantly different between SCI and BI patients, *X* ^2^ = 12.94, *d* *f* = 3, *P* =  .003. The mean disability score of SCI and randomly selected patients with BI based on the MRS is 4.36 ± SD: 0.1 and 3 ± SD: 0.6, respectively, in acute phase of stroke. Difference in mean disability at acute phase of stroke was not significant between two studied groups, *z* = 1.54, *P* =  .057. The mean changes of MRS for SCI and BI groups at 90 days later are 0.57 ± SD: 0.1 and 1.28 ± SD: 0.3, respectively. Difference in mean changes of disability was significant in two groups of patients, *z* = 2.65, *P* =  .019.  Table 1 demonstrates clinical characteristics of our SCI patients.

## 4. Discussion

SCI is a rare disease in our neurology division and constitutes 0.2% of cases with ischemic stroke. Sandson and Friedman described eight cases of SCI representing roughly 1.2% of all admissions for stroke in their center [5]. Although female gender was significantly more preponderant to SCI, the reason of these relationship is unknown. 66% of SCI patients reported by Zhang et al. were females [6]. There was also a predilection for women in reported cases of SCI by Hughes [7]. Mean age of patients with SCI is significantly lower than other ischemic patients in our study group. The main reason of this age difference could be distribution of etiologies in patients with SCI as discussed by Ghandehari [8]. Miscellaneous causes represented 50% of SCI etiologies in our SCI patients. It is well known that BI patients with miscellaneous etiologies have lower mean age than other BI patients [8]. Cheshire et al. evaluated 44 cases with SCI in the United States, and 67% of their cases have miscellaneous etiologies [9]. Iatrogenic etiologies constituted 21% of SCI causes in our case series and 30% of Cheshire et al. patients [9]. Due to the increased frequency of cardiovascular surgery and invasive interventional procedures, iatrogenic SCI is encountered more than it used to be. Paraplegia associated with unruptured aortic aneurysm repair was present in 5% of 101 patients who underwent surgery by Hollier et al. [10]. Spinal cord perfusion may be compromised by the aortic cross-clamping syndrome [9]. Aortic surgery was the etiology of SCI in 7% of our cases (number 3 in Table 1). A profound fall in perfusion pressure may lead to an ischemic myelopathy involving primarily the watershed area of midthoracic cord [2]. Sandson and Friedman reviewed 14 cases with SCI secondary to hypoperfusion [5]. Hypoperfusion was the mechanism of SCI in one of our cases (number 8 in Table 1) who underwent cardiac surgery. SCI is a well-known complication of epidural anesthesia in other case reports [11, 12], and we had one patient (number 1 in Table 1) with this complication. Neurosurgery especially in sitting position had been reported by Morandi as a cause of SCI [13] however none of our SCI developed after neurosurgery. SCI was observed in MRI of 48% of our SCI patients. The main reason of low rate of visible SCI in MRI of our cases is using lower generation 0.5 Tesla MRI equipment. However 1.5 Tesla or higher-generation MRI technologies may not also show SCI especially in the a few hours postevent [2, 6]. Anterior spinal artery syndrome constituted 85% of SCI presentations in our study group, which may be due to large territory of anterior spinal artery in the cord [1, 2]. SCI patients had more disability and less improvement at 90 days comparing to BI patients in our study. Similar results have been reported elsewhere [1, 2]. Pelser and van Gijn evaluated 10 SCI patients with mean followup period of 3 years [14]. Eight survived, and 2 died of cancer [14]. None of their cases had complete or good recovery of ischemic myelopathy. In Cheshire et al. case series, 24% of SCI patients had no improvement, the rest experienced some degree of functional improvement, and only 20% had a good recovery with minimal disability [9].

## 5. Conclusion

The spectrum of etiologies for SCI is diverse and includes its own set of susceptibilities according to the anatomic relationship of spinal cord vascularity to the spine and the aorta. The degree of recovery is generally poor in comparison with cerebral stroke where there is more likely to be adjacent neural tissue capable of assuming some of the lost function.

## Figures and Tables

**Table 1:** Clinical characteristics of 14 patients with spinal cord infarction.

Patient number	Age	Gender	Spinal cord syndrome	MRI^†^	Etiology
1	68	F	ASAS	−	Epidural anesthesia*$\hat{\;\;\;}$
2	26	F	ASAS	+	Uncertain
3	7	M	ASAS	−	Aortic surgery*$\hat{\;\;\;}$
4	61	F	ASAS	−	Cardioembolism
5	60	F	ASAS	−	Uncertain
6	35	M	ASAS	−	IV drug abuser*
7	56	M	ASAS	−	Cardiac surgery*$\hat{\;\;\;}$
8	55	F	ASAS	−	Aortic dissection*
9	18	M	TSCS	+	Arteriovenous malformation*
10	30	F	ASAS	+	Uncertain
11	28	F	ASAS	+	Uncertain
12	20	F	ASAS	−	Systemic lupus*
13	22	F	ASAS	+	Uncertain
14	58	M	Brown-Sequard	+	Atherosclerosis

ASAS: anterior spinal artery syndrome

TSCS: transverse spinal cord syndrome

* Miscellaneous etiologies

$\hat{\;}$ Iatrogenic causes

**^†^**+ and − mean the presence or absence of spinal cord infarction visible in MRI.
